# Supplementary material for: Polyacrylamide Bead Sensors for in vivo Quantification of Cell-Scale Stress in Zebrafish Development
Source: Sci Rep. 2019 Nov 19;9:17031. doi: 10.1038/s41598-019-53425-6 (PMC6864055; doi:10.1038/s41598-019-53425-6)
Supplement: Supplementary file 1 — Supplementary Information [file 41598_2019_53425_MOESM1_ESM.pdf]

Supplementary Information

Polyacrylamide Bead Sensors for *in vivo* Quantification of Cell-Scale Stress in Zebrafish Development

N. Träber[1,2*], K. Uhlmann[3*], S. Girardo[2], G. Kesavan[4], K. Wagner[2], J. Friedrichs[1], R. Goswami[4], K. Bai[4], M. Brand[4], C. Werner[1], D. Balzani[3], J. Guck[2,5]

[1] Leibniz-Institut für Polymerforschung Dresden e. V., Hohe Str. 6, 01069 Dresden, Germany

[2] Biotechnology Center, Center for Molecular and Cellular Bioengineering, Technische Universität Dresden, Tatzberg 47/49, 01307 Dresden, Germany

[3] Chair of Continuum Mechanics, Ruhr-Universität Bochum, Universitätsstraße 150, 44801 Bochum, Germany

[4] Center for Regenerative Therapies Dresden, Center for Molecular and Cellular Bioengineering, Technische Universität Dresden, Fetscherstr. 105, 01307 Dresden, Germany

[5] Max Planck Institute for the Science of Light, Staudtstraße 2, 91058 Erlangen

* These authors contributed equally to this work.

Correspondence:

Prof. Daniel Balzani (daniel.balzani@rub.de) and Prof. Jochen Guck (jochen.guck@mpl.mpg.de)

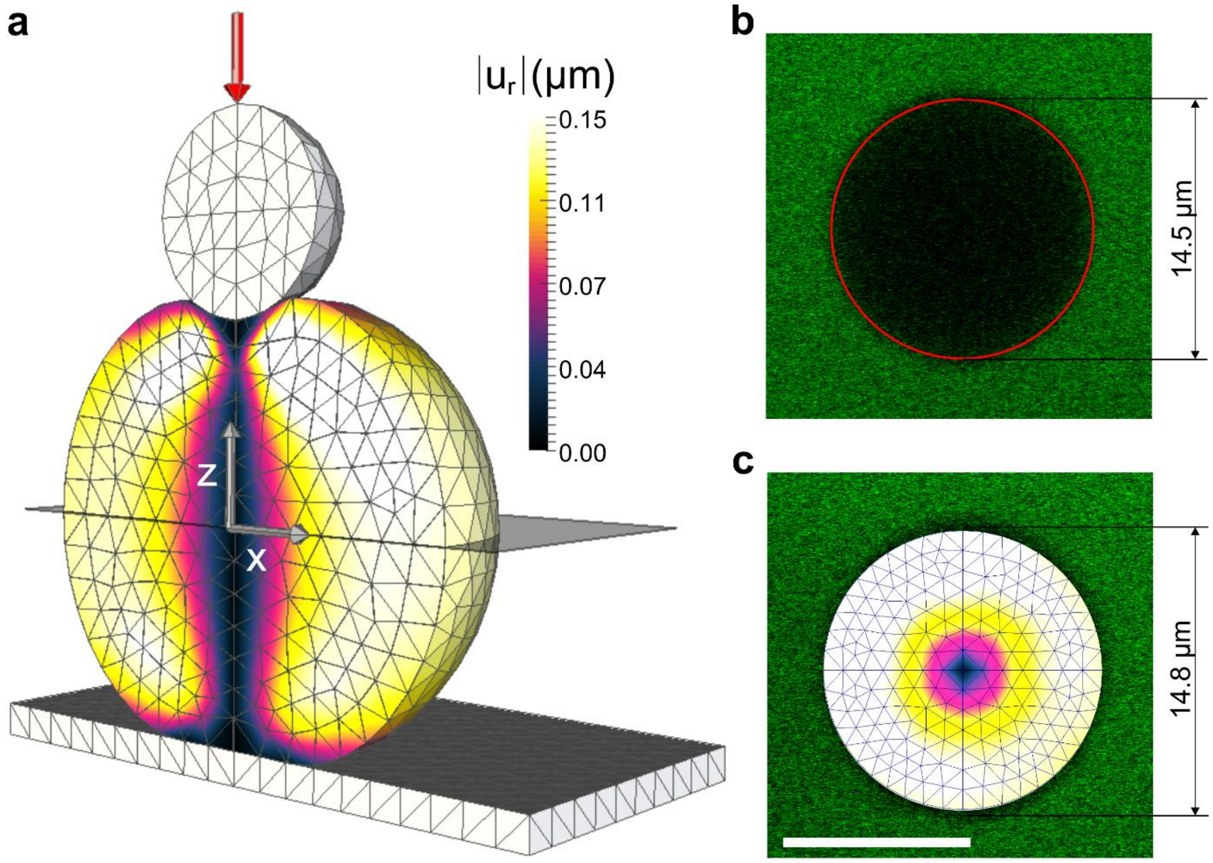

Supplementary Fig. 1: Numerical confirmation of Young´s modulus obtained with AFM indentation.

(a) Schematic of the AFM-based colloidal probe indentation process. An immobilized PAAm bead (diameter 14.5 µm) is indented with a spherical probe (diameter 5 µm) by a force of 10 nN. For the numerical simulation the measured Young's modulus of 2038.9 Pa of this particular PAAm bead was adopted. The contour plot visualizes the absolute values of displacement in x-direction (u_x). A numerical diameter change to 14.782 µm was obtained at $z = 0$ which was confirmed with confocal microscopy. **(b)** Confocal section of the undeformed PAAm bead in dextran-FITC solution (green). The red circle indicates the diameter of the bead. (**c**) Confocal section of the deformed PAAm bead in dextran-FITC solution (green) during AFM indentation with the contour plot of the radial displacements at the corresponding plane where $z = 0$. Scale bar, 10 µm.

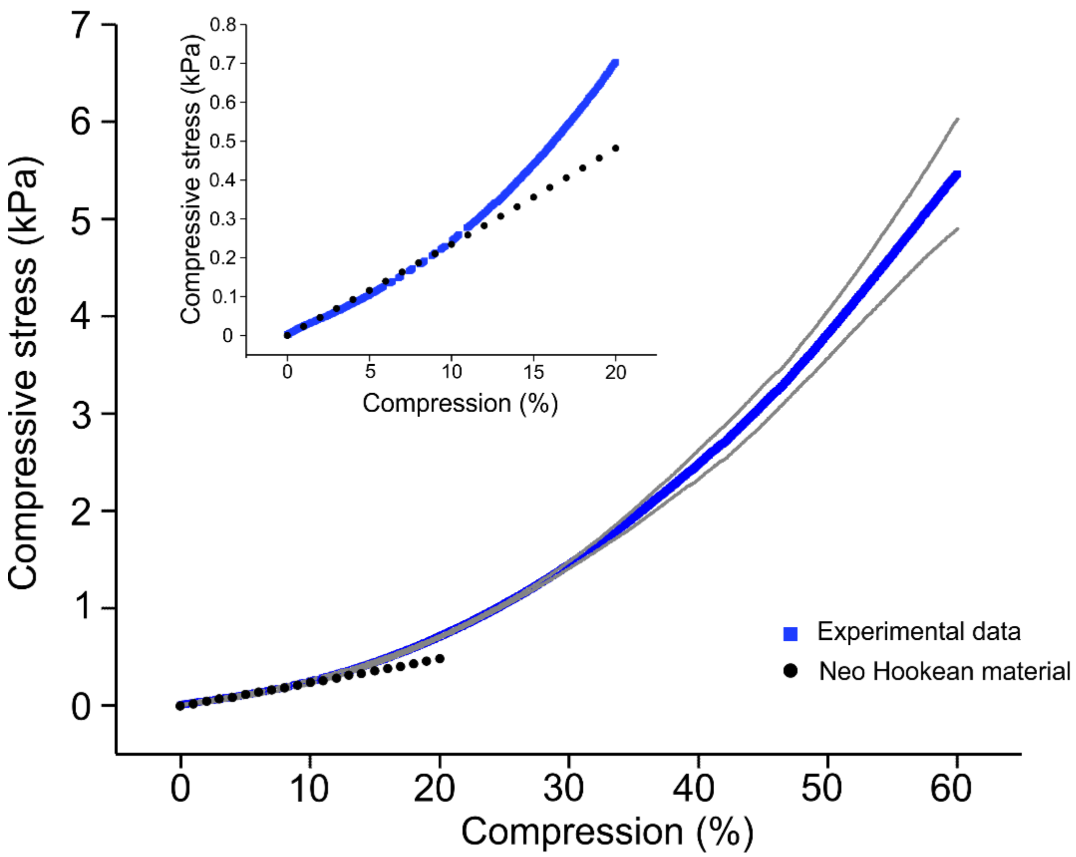

Supplementary Fig. 2: Plate rheometry of PAAm gels.

Stress-strain relation of PAAm gels (total monomer concentration 7.9%, $n = 3$) during compression. Mean values are shown as blue line, standard deviation is depicted in grey. Black dots illustrate numerical data obtained for an ideal Neo-Hookean material (with E = 2.3 kPa, Young´s modulus obtained for small strains). Inset: Enlarged view of the curves up to 20% compression.

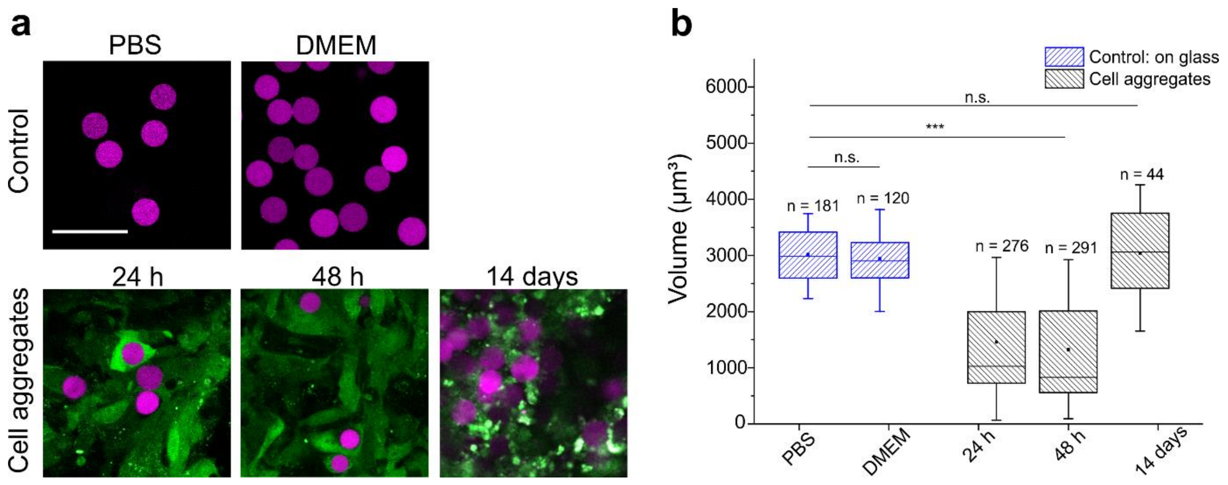

Supplementary Fig. 3: PLL-Cy3 PAA bead compression in MSC aggregates.

(a) Confocal microscopy images of PLL-Cy3 PAAm beads (magenta) in PBS and cell culture medium (DMEM), and during cultivation within human immortalized mesenchymal stromal cell (MSC) aggregates (green: GFP labeled cells). Scale bar, 50 µm. **(b)** Volume of PAAm beads during cultivation in MSC aggregates. Volume of PAAm beads on glass in PBS and in cell culture medium (DMEM) serve as control (blue boxes). Black boxes show the volume of the bead analyzed after 24 h, 48 h and 14 days of cultivation in MSC aggregates. After cell removal (14 days) the beads immediately return to their initial volume. The boxes are determined by the 25th and 75th percentiles, the whiskers represent standard deviation, the mean is indicated as filled square symbol and the median as straight line.

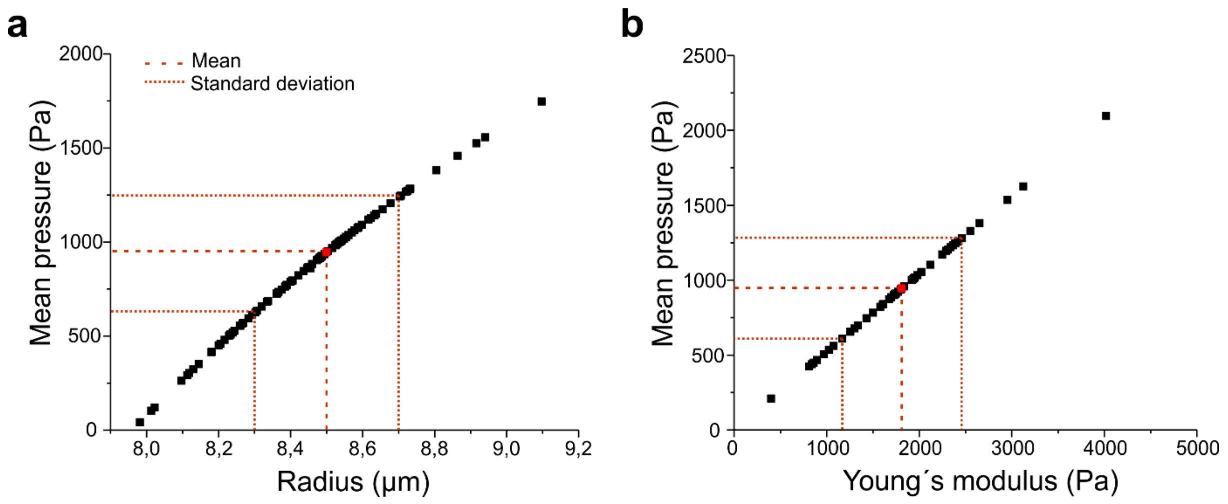

Supplementary Fig. 4: Uncertainty analysis of relevant parameters.

Load scenario of Fig. 3c (shear) was adopted to analyze the effect of uncertainty on two parameters: **(a)** Uncertainty analysis for varying radii of the undeformed bead and their impact on the resulting volumetric mean of the pressure in the bead. Representative samples of measured radii were adopted as simulation input. The Young’s modulus was set to the measured mean of 1813.86 Pa as fixed simulation input. The dashed red line indicates the corresponding mean and dotted lines represent the standard deviation. **(b)** Uncertainty analysis for varying Young’s moduli and their impact on the resulting volumetric mean of the pressure in the bead. The measured values of Young’s moduli were adopted as simulation input. The radius of the undeformed bead was set to the measured mean of 8.5 µm as fixed simulation input. The dashed red line indicates the corresponding mean and dotted lines represent the standard deviation.

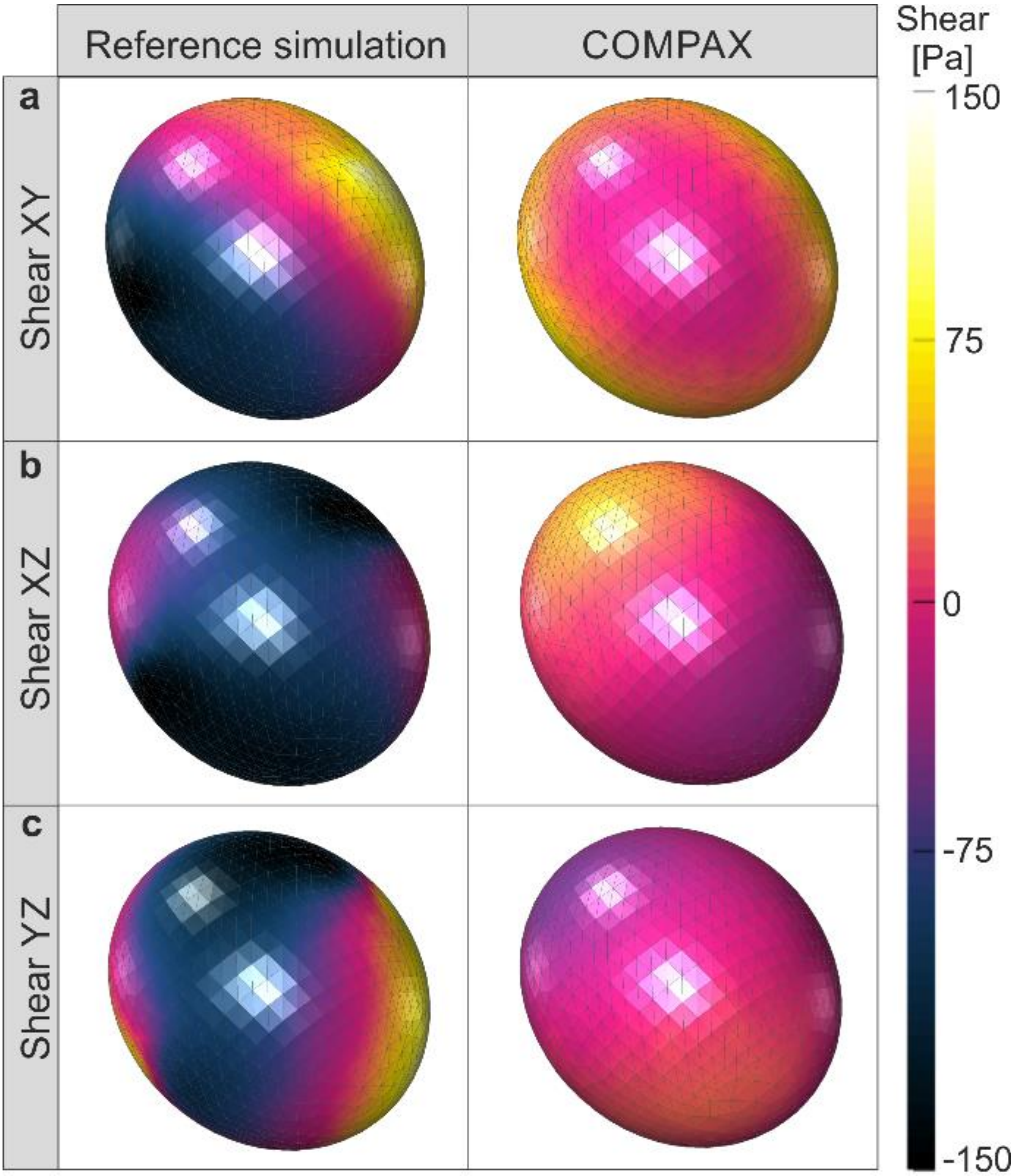

New Supplementary Fig. 5: Estimation of shear stresses reconstructed by COMPAX.

3D contour plots of Cauchy shear stresses in the reference simulation (left) and results of the COMPAX-method (right) for the load scenario shown in Fig. 3c. The alignment of the contour plots was chosen to match the orientation of the contour plots of pressure. The correct reconstruction of shear stresses is not possible without further improvements of the COMPAX-method.

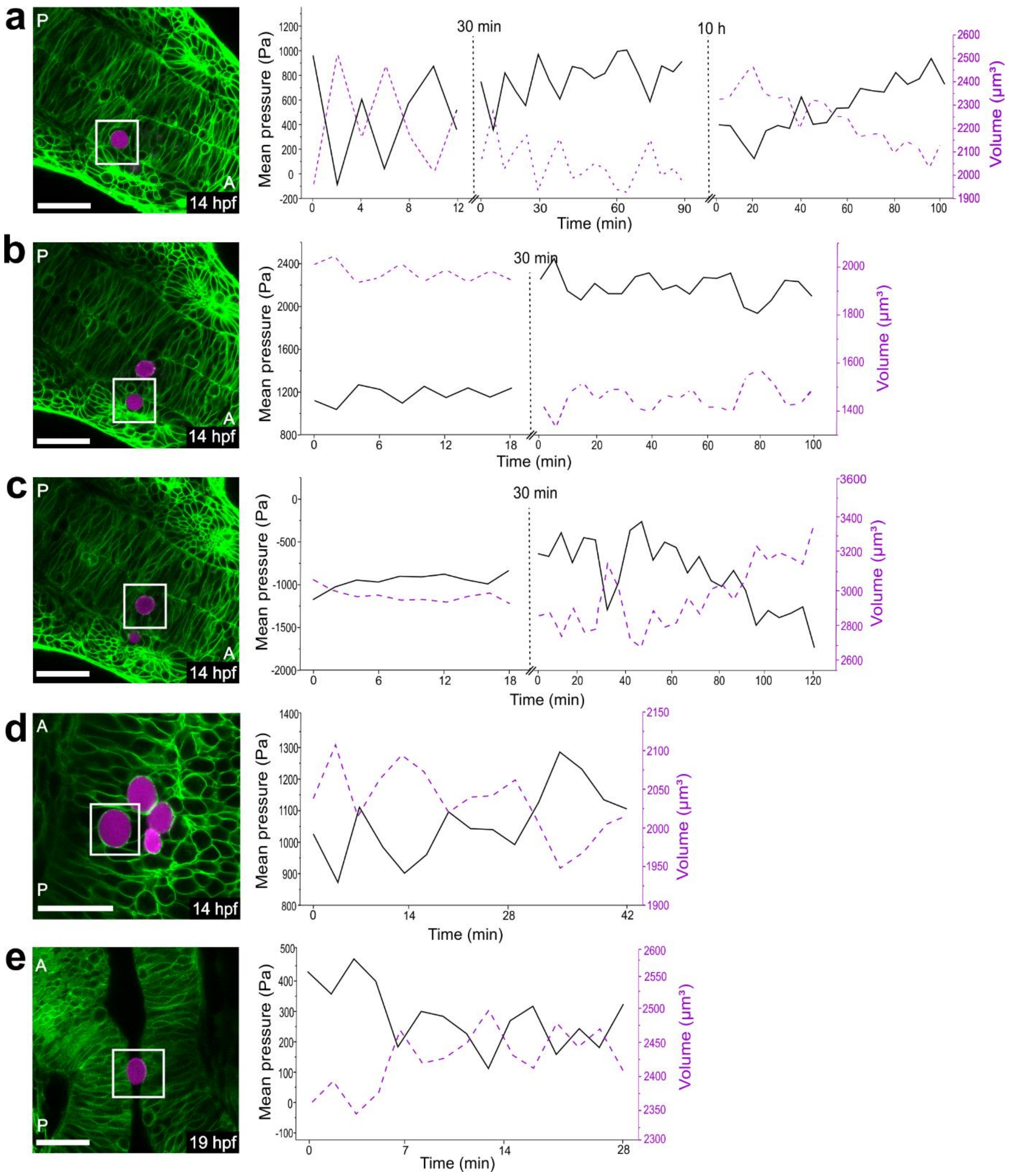

Supplementary Fig. 6: Temporal evolution of volumetric mean of pressure and bead volumes.

Volumetric mean of the pressure (black line) trends for **a)** the bead in the basal part of the neural tube (c.f., Fig. 5), **b)** the bead in the otic placode (c.f., Fig. 6a), **c)** the bead in the apical part of the neural tube (c.f., Fig. 6b), d) the bead at the midline of the neural tube (c.f. Fig. 6c) and **e)** the bead trapped in the neural tube (c.f. Fig. 6d). **(a) – (c)**: Left panel: confocal images always show the initial bead position. White boxes indicate the bead and anterior (A)-posterior (P) direction is always marked as well as the developmental stage. Scale bar, 30 µm. Right panel: dashed lines (magenta) show the corresponding volume of the PAAm bead for every time point.
